# New South Wales Dental Defence Association

**Published:** 1903-01

**Authors:** 


### NEW SOUTH WALES DENTAL DEFENCE ASSOCIATION.

One of the largest and most unanimous meetings of the dental profession which has taken place in the city of Sydney was held at the Manchester Unity Hall on Tuesday, November 11, to consider the advisability of taking steps to form an association for the purpose of mutual protection and support.

Sir James Graham, M.D., president of the Dental Board of New South Wales, occupied the chair, and was supported by Mr. Edward Reading and other members of the board. There were present, among other dental notables, Dr. Leopold Carter, Dr. I. P. Cliff, Dr. Stanley Rea, Mr. Donald Smith, Secretary of the Odontological Society.

The chairman said that it was a matter of history that this state was the last part of the civilized world to realize the enormous risks which were run in allowing the profession of dentistry to get into the chaotic state that existed until quite recently. The passing of a measure through Parliament gave the public some guarantee of a change in this condition. But the dental board brought into existence by that act had no function and no jurisdiction but that which was provided for it within the four corners of the measure. The time was not far distant when the constitution of the board would be placed in the hands of the dentists themselves. The legislation, however, useful as it has been, was no adequate guarantee to the public, and it was hoped that before long Parliament would be induced, in the interests of the people, to amend the law so as to further guarantee the professional qualities of the dentists registered under its provisions. This meeting was called for the purpose of establishing an association having the interests of the public in view, and for the purposes of professional interests. It was known that dentists and other professional men ran certain risks, which resulted in moral and material damage. Men who were exposed to these dangers recognized that only by organization they could successfully combat them. In no sense would the institution be a trade union, but simply an institution to defend the members against loss and hardship.

Mr. Horace Taylor (secretary *pro tem.*) explained the steps that had been taken to bring the movement under the notice of the profession, and exhibited some five hundred letters he had received from all parts of the state in congratulation and support of the proposal.

Mr. Ernest Blackwell moved "That this meeting is of opinion that it is desirable to form a Dental Defence Association, having for its objects—

A. To render assistance to any member who may be threatened with, or involved in any action at law or prosecution arising out of the practice of his profession within this state.

B. To assist in any movement which will promote fraternity among the members, or elevate and improve the social and legal status of dentists.

C. Promotion of the welfare of the dental profession of New South Wales. He pointed out that similar institutions existed in all the other Australian states and in other professions in this state.

Mr. Charles Hodgson, in seconding the resolution, stated that such an institution would have his heartiest support. He had advocated its establishment for years, and pointed out the number of speculative actions which had of late been brought against dentists, in which it was found that, in consequence of the impecuniosity of the complainants, the defending dentists, although adjudged to be without blame, were, notwithstanding, ruined through having to pay their own costs. If these cases had been met with determination at the outset by a powerful association like the one now proposed, these speculative actions would seldom if ever be brought.

In the discussion much light was thrown upon the practice of blackmail and other evils affecting the dental profession that are going on, and it was shown that dentists often paid lump sums to square unjust claims rather than run the risk of getting their costs in a law-suit.

The resolution was adopted without dissent.

Dr. Henry Peach moved "That a committee of twelve be appointed to draw up a scheme by which these objects may be attained and report at a future meeting."

The resolution was adopted, the voting resulting in the return of the following gentlemen as members of the committee: Henry Peach, D.D.S., E. G. Moon, Charles Hall, C. C. Marshall, Ernest Blackwell, W. R. Fitzsimons, Stanley Rea, D.D.S., J. E. Forsyth, D.D.S., I. P. Cliff, D.D.S., F. P. Head, Walter Robinson, and Edward Reading.

